# Expression of phospho-ERK1/2 and PI3-K in benign and malignant gallbladder lesions and its clinical and pathological correlations

**DOI:** 10.1186/1756-9966-28-65

**Published:** 2009-05-18

**Authors:** Qinglong Li, Zhulin Yang

**Affiliations:** 1 Research Laboratory of Hepatobiliary Diseases, The Second Xiangya Hospital, Central South University, Changsha, Hunan 410011, PR China

## Abstract

**Background:**

An increasing number of studies have shown that ERK and PI3-K/AKT signaling pathways are involved in various human cancers including hepatocellular carcinoma and cholangiocarcinoma. However, few studies have examined gallbladder cancer specimens, and little is known about the clinical and pathological significance of ERK1/2 and PI3-K/AKT signaling changes in gallbladder adenocarcinoma. In this study, we examined phospho-ERK1/2 (p-ERK1/2) and PI3K expression and analyzed its clinicopathological impact in gallbladder adenocarcinoma.

**Methods:**

Immunohistochemistry was used to detect and compare the frequency of p-ERK1/2 and PI3-K expression in gallbladder adenocarcinoma, peri-tumor tissues, adenomatous polyps, and chronic cholecystitis specimens.

**Results:**

The positive staining for p-EKR1/2 and PI3-K were 63/108 (58.3%) and 55/108 (50.9%) in gallbladder adenocarcinoma; 14/46 (30.4%) and 5/46 (10.1%) in peri-tumor tissues; 3/15 (20%) and 3/15 (20%) in adenomatous polyps; and 4/35 (11.4%) and 3/35 (8.6%) in chronic cholecystitis. The positive rate of p-ERK1/2 or PI3-K in gallbladder adenocarcinoma was significantly higher than that in peri-tumor tissue (both, *P *< 0.01), adenomatous polyps (p-ERK1/2, *P *< 0.01; PI3-K, *P *< 0.05), and chronic cholecystitis (both, *P *< 0.01). The positive staining for p-ERK1/2 or PI3-K was significantly lower in well/highly-differentiated adenocancinomas with maximal diameter < 2.0 cm, no metastasis to lymph node, and no infiltration of regional tissues or organs compared to poorly-differentiated adenocarcinomas which are characterized by a maximal diameter ≥ 2.0 cm, with metastasis to lymph node and infiltration of regional tissues or organs (*P *< 0.05 or *P *< 0.01). Moreover, the frequency of p-ERK1/2 expression in gallbladder adenocarcinomas without gallstone was significantly lower than those with gallstones. Increased expression of p-ERK1/2 (*P *< 0.05) and PI3K (*P *= 0.062) was associated with decreased overall survival. Multivariate Cox regression analysis showed that increased p-ERK1/2 expression was an independent prognostic predictor in gallbladder carcinoma (*P *= 0.028).

**Conclusion:**

Increased expression of p-ERK1/2 and PI3K might contribute to gallbladder carcinogenesis. p-ERK1/2 over-expression is correlated with decreased survival and therefore may serve as an important biological marker in development of gallbladder adenocarcinoma.

## Background

Gallbladder cancer is a relatively rare but terminal malignancy occurring predominantly in elderly women. It accounts for nearly two-thirds of biliary tract cancers, making it the most common primary biliary cancer and the fifth most common cancer of the gastrointestinal tract [1,2]. More than 85% of gallbladder cancers belong to adenocarcinomas that are often well or moderately differentiated, and the remaining 15% are squamous, adenosquamous or undifferentiated carcinomas. Surgery is the only recommended treatment currently available. However, more than 70% of cases are un-resectable due to local invasion into critical structures or metastasis beyond regional confines. A better understanding of pathological molecular mechanisms of gallbladder carcinogenesis may provide insights for developing novel targeted therapies for this deadly disease.

The development of cancer in man involves multiple genetic changes that often lead to dysfunction of certain signaling pathways controlling cell fate, cell growth, and cell survival or cell death. Activation of the extracellular signal-regulated kinase (ERK) 1/2 and PI3-K signaling pathways is believed to be involved in the pathological processes of cancer development. Activation of the ERK1/2 pathway results in cell proliferation [3,4] and leads to malignant transformation both *in vitro *and *in vivo *[5,6], and activation of the PI3-K/AKT signaling pathway inhibits apoptosis and promotes cell survival. An increasing number of studies have shown that both ERK and PI3-K/AKT signaling pathways are over-activated in various human cancers including breast cancer, lung cancer, colorectal cancer, pancreatic cancer, malignant melanoma, hepatocellular carcinoma, and cholangiocarcinoma [6-9]. In hepatocellular carcinoma, activation of ERK1/2 indicates aggressive tumor behavior and constitutes an independent prognostic factor. Increased p-ERK1/2 and p-AKT levels correlate with decreased overall survival [10]. Elevated p-ERK1/2 and p-AKT expressions have also been found in cholangiocarcinoma [7]. Both EKR1/2 and AKT can be activated by a number of factors including EGFR, inflammation signals mediated by cytokine receptors, mutation of oncogenes such as Ras and Raf, and bile acids [8].

Since few studies have examined gallbladder cancer specimens [11], little is known about the clinical or pathological significance of ERK1/2 and PI3-K/AKT signaling changes in gallbladder adenocarcinoma. In this study, we examined the frequency of p-ERK1/2 and PI3K expression in gallbladder adenocarcinoma specimens by means of immunohistochemistry and attempt to elucidate the clinical and pathological significance of changes in the p-ERK1/2 and PI3-K/AKT pathways in gallbladder adenocarcinoma.

## Methods

### Materials

108 gallbladder carcinoma specimens were collected from the First and Second Xiangya hospitals affiliated to Central South University, and People's Hospital of Hunan Province, Changsha, China. 77 (71.3%) specimens came from female patients and 31 males (28.7%). All specimens were diagnosed as adenocarcinomas, of which 9 had adenoma lesions, 29 were highly differentiated, 29 moderately differentiated, 30 poorly differentiated, and the remaining 11 were mucous adenomas (10.2%). During surgery, 59 cases (54.6%) were found to have invasion of peri-cholecystic tissues and organs, 59 cases (54.6%) demonstrated local lymph node metastases; and 58 cases (53.7%) had evidence of gallstones/cholelithiasis. The applied surgical modalities include radical resection in 34 cases (31.5%), palliative resection/operation in 48 cases (44.4%), and 26 cases (24.1%) unresectable due to local invasion into critical structures or metastasis beyond regional confines. From the above 108 gallbladder adenocarcinoma samples, we obtained the peri-tumor tissues from 46 case (distance to adenocarcinomas ≥3 mm), 10 of which were normal by pathological analysis. Mild, moderate or severe atypical proliferation was observed in 10, 12 and 14 cases, respectively. 15 specimens of gallbladder adenoma polyps were obtained from the Second Affiliated Hospital of Central South University (including 10 female and 5 male, average age 52 years old, range 42 to 60 years). The polyploidy adenomas ranged from 0.08 – 15 mm in size, 5 out of the 15 had moderate to severe proliferation. In addition, 35 chronic cholecystitis specimens were obtained (15 with chronic cholecystitis alone, 20 with chronic cholecystitis and gallstones) as controls. Histologically, the 35 specimens included 11 with normal gallbladder mucosa, 12 with mild atypical proliferation, 7 with moderate atypical proliferation, and 5 with severe atypical proliferation. All the above samples were fixed in 4% formalin, and 4 micron sections were prepared for immunohistochemistry studies.

### Immunohistochemistry

For p-ERK1/2 and PI3-K detection, immunostaining was carried out using EnVision™ (ChemMate™EnVison +/HRP/DAB, Rabbit/Mouse Two Step Staining Method) according to the manufacture's protocol (DAKO laboratories Inc, California, USA). Briefly, paraffin-embedded gallbladder adenocarcinoma tissues were cut into 4 μm thick sections. The sections were de-paraffinized and incubated with 3% of H_2_O_2 _solution for 15 min, followed by EDTA-trypsinase digestion (0.125%, pH 9.0) for 15 min, then soaked with PBS (pH7.4) 3 times, each for 5 minutes. The pre-treated sections were then incubated with rabbit anti-human p-ERK1/2 or PI3-K (Bosite Inc, Wuhan, China) for 60 min at room temperature. Solution A (ChemMate™EnVison +/HRP) was added and incubated for another 30 min. Substrate DAB liquid was added and followed by hematoxylin counter-staining. Slides were dehydrated with different concentrations of alcohol and soaked in xylene for 5 minutes (3 times), and then mounted permanently with neutral balsam. Slides were examined independently by two pathologists. The results of p-ERK1/2 or PI-3K immunostaining were considered to be positive when more than 25% of the tumor cells were stained. The positive controls were provided by Bosite Inc, Wuhan.

### Statistical analysis

The SPSS13.0 program was used for calculation of interrelationships between the analyzed p-ERK1/2 or PI3-K and histological or clinical factors by χ^2 ^independence test. Fisher's exact probability test was also used for analyzing statistical association between the two independent sample groups. The results were considered to be significant when the *P *value were less than 0.05. Disease specific overall survival analyses were determined and compared using the Kaplan-Meier method and the log-rank test. For multivariate analysis the Cox regression method was performed. 95% confidence intervals were used overall.

## Results

### Expression of p-ERK1/2 and PI3-K in human gallbladder adenocarcinoma, peri-tumor tissues, adenomatous polyps, and chronic cholecystitis

**Figure 1:**
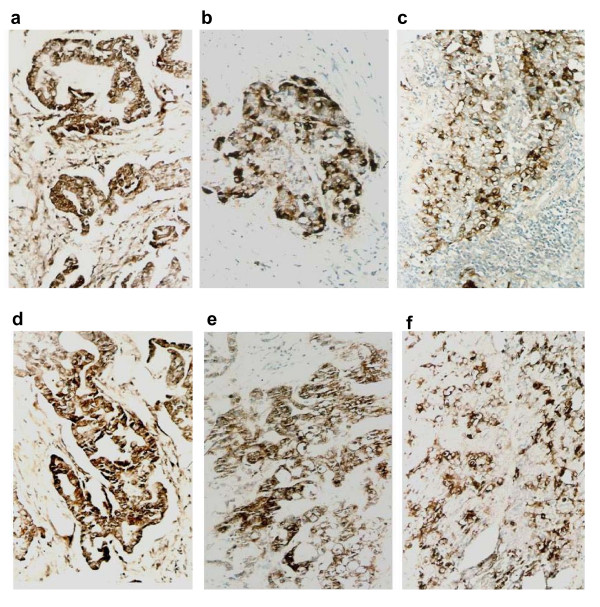
**Expression of p-ERK1/2 and PI3-K (original magnification 200×)**. Expression of p-ERK1/2 in well-differentiated gallbladder adenocarcinoma (a), moderately-differentiated gallbladder adenocarcinoma (b), and poorly-differentiated gallbladder adenocarcinoma (c); PI3-K expression in well-differentiated (d), moderately-differentiated (e), and poorly-differentiated gallbladder adenocarcinoma (f).

**Figure 2:**
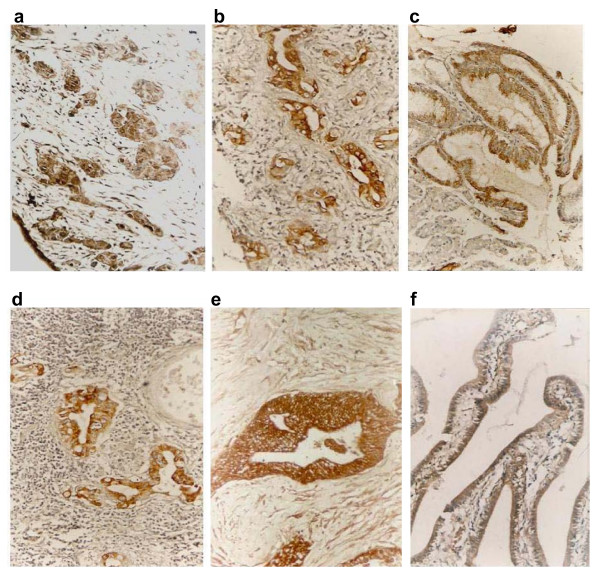
**Immunohistochemical staining of p-ERK1/2 and PI3-K (original magnification 200×)**. p-ERK1/2 expression in peri-tumor tissues with severe atypical proliferation of gallbladder adenocarcinoma (a), in gallbladder adenoma polyps with moderate atypical proliferation (b), in chronic cholecystitis with moderate atypical proliferation (c). PI3-K staining in surrounding tissues with severe atypical proliferation of gallbladder adenocarcinoma (d), in gallbladder adenoma polyps with severe atypical proliferation pericancerous tissues (e), and in chronic cholecystitis with mild (f).

Immunohistochemistry for p-ERK1/2 and PI3-K were conducted with 108 gallbladder adenocarcinomas, 46 surrounding tissues of gallbladder adenocarcinoma, 15 adenoma polyps, and 35 chronic cholecystitis samples. Positive staining for p-ERK1/2 was observed in the cytoplasm and/or nucleus (Figure 1 and 2a–c). PI3-K staining was mostly seen in the cytoplasm as expected (Figure 1 and 2d–f). As shown in Table 1, of the 108 gallbladder adenocarcinomas, expression of p-ERK1/2 and PI3-K was detected in 63 (58.3%) and 55 cases (50.9%), respectively. In the 46 surrounding tissues of gallbladder adenocarcinoma, p-ERK1/2 and PI3-K were positive in 14 (30.4%) and 5 (10.1%) cases, respectively. Moderate to severe atypical hyperplasia were observed in all gallbladder mucous epithelium in p-ERK1/2 positive cases. In PI3-K positive samples, however, gallbladder mucous epithelium was normal in one case, mild atypical hyperplasia in one case, while moderate and severe atypical hyperplasia were seen in one and 2 cases, respectively. Positive staining for p-ERK1/2 and PI3-K was both observed in 3 out of 15 adenoma polyps which all showed moderate to severe atypical hyperplasia. In the chronic cholecystitis group, p-ERK1/2 and PI3-K staining was positive in 4 (11.4%) and 3 (8.6%) of the 35 cases, respectively. Gallbladder mucous epithelium in positive specimens showed moderate to severe atypical hyperplasia. Overall, the frequency of samples positive for p-ERK1/2 and PI3-K in gallbladder adenocarcinomas was significantly higher than that in surrounding tissues (χ^2^_pERK _= 10.04, *P *< 0.01; χ^2^_PI3-K _= 21.77, *P *< 0.01), in adenoma polyps (χ^2^_pERK _= 7.78, *P *< 0.01; χ^2^_PI3-K _= 5.06, *P *< 0.01), and in chronic cholecystitis (χ^2^_pERK _= 23.35, *P *< 0.01; χ^2^_PI3-K _= 19.67, *P *< 0.01).

**Table 1:** Expression of p-ERK1/2 and PI3-K in gallbladder adenocarcinoma, surrounding tissues, adenomatous polyps, and chronic cholecystitis as determined by immunohistochemistry.

Disease type	Total	pERK1/2 + (%)	P value	PI3-K + (%)	P value
Gallbladder adenocarcinoa	108	65/108 (58.3%)	<0.01	55/108 (50.9%)	<0.01
Surrounding tissues	46	14/46 (30.4%)		5/46 (10.1%)	
Adenomatous polyps	15	3/15 (20%)		3/15 (20%)	
Chronic cholecystitis	35	4/35 (11.4%)		3/35 (8.6%)	

### Correlation of p-ERK1/2 and PI3-K expression with clinical and pathological features of gallbladder adenocarcinoma

We further analyzed the correlation of p-ERK1/2 and PI3-K expression with the clinical and pathological features of gallbladder adenocarcinoma. As shown in Table 2, the frequency of samples staining positive for p-ERK1/2 and PI3-K in cases with small tumor size (<2 cm in diameter), without lymph node metastasis, and no invasion of surrounding tissues was significantly lower than in cases with larger tumor size (>2 cm), lymph node metastasis, and invasion in surrounding tissues (*P *< 0.05 or *P *< 0.01). Interestingly, the positive staining for p-ERK1/2 in cases concomitant with gallstones/cholelithiasis was significantly higher than in cases without gallstones (*P *< 0.05). Moreover, positive staining for p-ERK1/2 and PI3-K in adenoma or well-differentiated adenocarcinomas was significantly lower compared to poorly-differentiated adenocarcinomas as shown in Table 3 (both, *P *< 0.01).

**Table 2:** Expression of p-ERK1/2 and PI3-K as determined by immunohistochemistry, and clinicopathological variables in 108 patients with gallbladder adenocarcinoma.

Group	Total	pERK1/2 + (%)	P value	PI3-K + (%)	P value
Sex					
Female	77	47 (61.0)	>0.05	41(53.2)	>0.05
Male	31	16(51.6)		14(45.2)	
					
Age					
≤45	24	12(50)	>0.05	12(50)	>0.05
>45	84	51(60.7)		43(51.2)	
					
Tumor diameter					
<2.0 cm	31	13(41.9)	<0.05	11(35.5)	<0.05
≥2.0 cm	77	50(64.9)		44(57.1)	
					
Lympho node metastasis					
No	49	20(40.8)	<0.01	16(32.7)	<0.01
Yes	59	43(72.9)		39(66.1)	
					
Surrounding tissue invasion					
No	49	21(42.9)	<0.01	17(34.7)	<0.01
Yes	59	42(71.2)		38(64.4)	
					
Gallstones					
No	50	24(48.0)	<0.05	22(44)	>0.05
Yes	58	36(67.2)		33(56.9)	

**Table 3:** p-ERK1/2 and PI3-K expression in gallbladder adenocarcinoma as determined by immunohistochemistry.

	Total	pERK1/2 + (%)	P value	PI3-K + (%)	P value
Pathology type*					
Adenoma canceration	9	3(33.3)		2(22.2)	
Well-differentiated	29	12(41.4)	<0.01	10(34.5)	<0.01
Moderately-differentiated	29	18(62.1)		16(55.2)	
Poorly-differentiated	30	25(83.3)		23(76.7)	
Mucous adenoma	11	5(45.5)		4(36.4)	

*Comparison of adenoma lesions and poorly-differentiated adenocarcinomas, *P*_pERK _= 0.08, *P*_PI3-K _= 0.05, comparison of the well-differentiated and poorly-differentiated, χ^2^_pERK _= 11.10, *P *< 0.01; χ^2^_PI3-K _= 10.65, *P *< 0.01.

### Correlation of p-ERK1/2 and PI3K expression with disease specific overall survival

**Figure 3:**
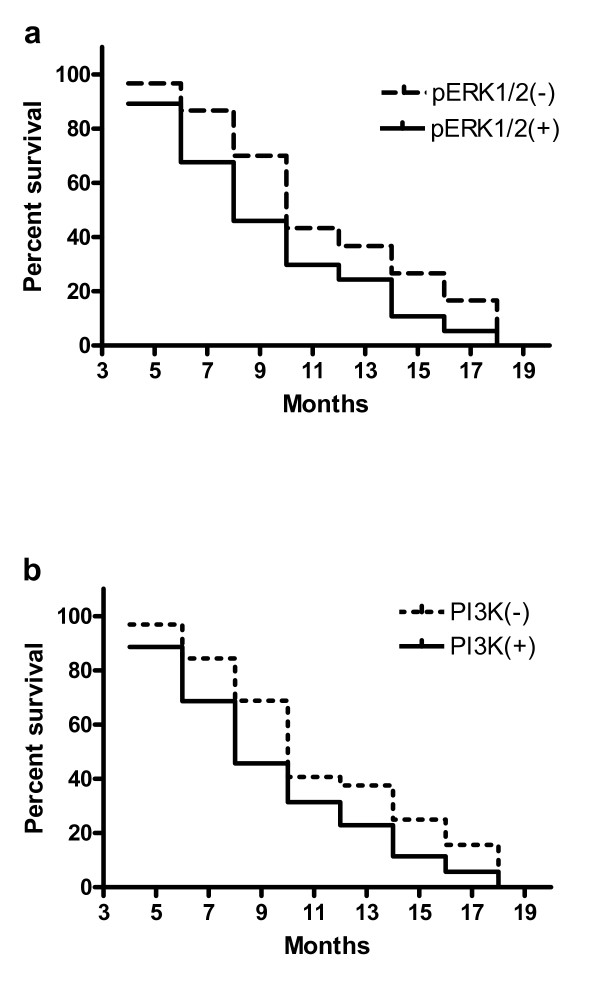
**Kaplan-Meier plots for overall survival in 67 patients with gallbladder carcinoma surgery in relation to p-ERK1/2 and PI3K expression**. (a) Positive or increased p-ERK1/2 expression was associated with reduced over survival (P = 0.045, log rank test). (b) Increased PI3K expression was also related to reduced overall survival (P = 0.062, log rank test).

After surgical resection, only 67 out of 108 patients were able to be followed up via phone or mail surveys. Of the 67 cases with follow-up, 20 cases had over one year survival, and 47 died within one year after surgery, with a mean survival time of 9.6 ± 5.2 months. 37 of the 67 (55.2%) patients had positive immunohistochemical staining of p-ERK1/2, and 35 (52.2%) had positive PI3K staining. The relevance of positive p- ERK1/2 and PI3K expression to patients survival was examined by univariate Kaplan-Meier survival analysis. Overall survival was inversely associated with positive or increased expression of p-ERK1/2 (*P *= 0.045) (Figure 3a) and PI3K (*P *= 0.062) (Figure 3b). The relevance of overall survival and other clinical pathological characteristics were also assessed by univariate analysis which showed that the overall survival was associated with tumor pathological type (*P *= 0.031), tumor diameter (*P *= 0.003), lymph node metastasis (*P *= 0.005) and surrounding tissue invasion (*P *= 0.002). All factors that showed significant association in the univariate Kaplan-Meier analysis were subsequently subject to multivariate Cox regression survival analysis, which indicated that lymph node metastasis and surrounding tissue invasion were the most significant predictors of short overall survival, followed by p-ERK1/2 over-expression (Table 4).

**Table 4:** Multivariate Cox regression analysis of overall survival in 67 patients with surgical resection of gallbladder carcinoma.

Group	Category	SE(B)	*P*	95% CI for Exp(B)	
				
				Inferior	Superior
Pathology type	Adenoma canceration/well-/moderately-/poorly-differentiated/mucous adenoma	1.73	0.249	0.82	2.15
Tumor diameter	<2.0 cm/≥2.0 cm	2.08	0.041	1.01	3.99
Lympho node metastasis	No/Yes	2.58	0.019	1.21	3.97
Surrounding tissue invasion	No/Yes	2.46	0.025	1.17	3.86
p-ERK1/2	-/+	2.35	0.028	1.07	4.19
PI3K	-/+	2.24	0.037	1.03	4.03

SE = Standard Error, B = Beta, CI = Confidence Interval.

## Discussion

In the present study, we examined p-ERK1/2 and PI3-K expression by immunohistochemistry in 108 human gallbladder adenocarcinoma samples from separate individuals. 58.3% and 50.9% of the specimens showed strong positive staining for p-ERK1/2 and PI3-K, respectively, indicating that both p-ERK1/2 and PI3-K/AKT might be potential biomarkers of gallbladder cancer. Compared to benign lesions and peri-tumor tissues, positive staining for p-ERK1/2 and PI3-K in gallbladder adenocarcinoma was significantly higher. Expression of p-ERK1/2 and PI3-K was correlated with a low grade of differentiation in adenocarcinoma (Table 1). Moreover, p-ERK1/2 and PI3-K staining was more frequently detected in gallbladder adenocarcinoma cases with larger tumor size, lymph node metastasis, and surrounding tissue invasion compared to cases with smaller tumor size, without metastasis or tissue invasion (Table 2). These findings suggest that activation of both the p-ERK1/2 and PI-3K/AKT signaling pathways might be involved in malignant transformation and progression of gallbladder adenocarcinoma. On multivariate analysis, there was a significant association between p-ERK1/2 over-expression and reduced survival (Table 4).

To our knowledge, this is the first report showing a correlation of p-EKR1/2 and PI3-K expression with clinical and pathological features, including tumor size, lymph node metastasis and surround tissue invasion. Hori et al [11] demonstrated that 77% of extra-hepatic biliary tract cancer showed positive staining for p-MAPK and 47% for p-AKT. However, those results showed no positive correlation between p-MAPK/p-AKT expression and clinical and pathological features, including tumor stage and pT category in extra-hepatic biliary tract cancer. The study performed by Hori et al was based on a small cohort with 30 patients including 15 with gallbldadder cancer, 13 with bile duct cancer and 2 with ampullary cancer. Another study by Wu et al. also revealed elevated level of p-AKT in 74.1% (20 of 27) of human gallbladder cancer specimens [12]. A number of other studies showed similar positive rates of expression of p-MAPK/p-ERK1/2 or p-AKT in cholangiocarcinoma [7], intra-hepatic cholangiocarcinoma [8], and cholangiocarcinoma [13], but the association with clinical and pathological features remain inconclusive. Javle et al. demonstrated that expression of p-AKT may be associated with improved survival [13]. However, in another study Schmitz et al. showed that neither p-ERK1/2 nor p-AKT expression had an impact on patients survival in a larger and more homogenous cohort of solely intra-hepatic cholangiocarcinoma [8].

ERK1/2 and PI3-K signaling pathways are associated with cell proliferation, transformation and survival. The exact molecular mechanism in which ERK1/2 and/or AKT remains constitutively activated in a variety of human cancers is however not well understood. EGFR activation triggers multiple signaling cascades which include MAPK/ERK1/2 and PI3-K/AKT pathways, resulting in cell proliferation, differentiation, angiognenesis, metastasis, and inhibition of apoptosis [14,15]. Over-expression of EGFR was found in patients with malignancies of gallbladder, ampullary and common bile duct [16-19]. Somatic mutations of EGFR in the tyrosine kinase domain have been identified in a subgroup of patients with cholangiocarcinoma or gallbladder carcinoma [15]. The mutations lead to sustained activation of signaling and results in cell survival and proliferation. Mutations of oncogenes have also been identified in cholangiocarcinoma. For example, K-Ras and B-Raf mutations were found in 22% and 45% of cholangiocarcinoma, respectively [20]. Chronic inflammatory condition caused by gallstone or cholecystitis has also been linked to the development of gallbladder cancer[21,22]. How chronic inflammation contributes to gallbladder cancer and how inflammatory factors affect EKR1/2 and PI-3K/AKT pathways in gallbladder cells is yet to be explored. Several reports show that cholangiocarcinoma cells constitutively secrete IL-6 which may activate ERK1/2 and AKT [23-25]. In our study, 58 of the 108 (54%) patients had gallstones. Interestingly, activated EKR1/2 but not PI3-K is correlated with presence of cholelithiasis (Table 2). The underlying mechanism needs to be further studied.

Cross-talk between the ERK1/2 and PI3-K signaling pathways has been implied at different stages of cholangiocarcinoma and extrahepatic biliary tract cancers [11]. Our study also indicates that there is a positive correlation between the frequency of p-ERK1/2 and PI3-K expression, suggesting a possible cross-talk of the two pathways in gallbladder adenocarcinoma. Further studies to address the underlying mechanisms in which activation of the ERK and AKT pathways contributes to increased tumor aggressiveness and progression in gallbladder adenocarcinoma might offer the possibility to utilize serine/threonine kinase inhibitors as targeted therapeutics.

## Conclusion

Our study revealed that the frequency of p-ERK1/2 and PI3-K expression is increased in gallbladder adenocarcinoma. Activation of ERK1/2 and PI3-K signaling pathways is correlated with decreased patients' survival. ERK1/2 and PI3-K pathways may serve as new targets for furture development of novel treatments for gallbladder adenocarcinoma.

## Competing interests

The authors declare that they have no competing interests.

## Authors' contributions

The two authors contributed equally to the research work and writing of the manuscript.
